# *Pelargonium sidoides* root extract for the treatment of acute cough due to lower respiratory tract infection in adults: a feasibility double-blind, placebo-controlled randomised trial

**DOI:** 10.1186/s12906-021-03206-4

**Published:** 2021-01-29

**Authors:** Merlin Willcox, Catherine Simpson, Sam Wilding, Beth Stuart, Dia Soilemezi, Amy Whitehead, Alannah Morgan, Emma Wrixon, Shihua Zhu, Guiqing Yao, Fran Webley, Ruiyang Yan, Jennifer Bostock, Margaret Bell, Gareth Griffiths, Geraldine Leydon, Paul Little, Christopher Butler, Alastair D. Hay, Michael Moore

**Affiliations:** 1 School of Primary Care, Population Sciences and Medical Education, University of Southampton, Aldermoor Health Centre, Aldermoor Close, Southampton, SO16 5ST UK; 2 Southampton Clinical Trials Unit, University of Southampton and University Hospital Southampton NHS Foundation Trust, Southampton, UK; 3 Department of Health Sciences, University of Leicester, Leicester, UK; 4 Nuffield Department of Primary Care Health Sciences, University of Oxford, Oxford, UK; 5 Centre for Academic Primary Care, Bristol Medical School, Population Health Sciences, University of Bristol, Bristol, UK

**Keywords:** Feasibility clinical trial, Double-blind randomised, Placebo-controlled, Acute bronchitis, Cough, Herbal medicine, Retention, Cluster-randomised

## Abstract

**Background:**

*Pelargonium sidoides* DC (Geraniaceae) root extract, EPs®7630 or “Kaloba®”, is a widely used herbal remedy for respiratory infections, with some evidence of effectiveness for acute bronchitis. However, it is not yet widely recommended by medical professionals in the UK. There is a need to undertake appropriately designed randomised trials to test its use as an alternative to antibiotics. The aim was to assess the feasibility of conducting a double-blind randomised controlled trial of *Pelargonium sidoides* root extract for treatment of acute bronchitis in UK primary care, investigating intervention compliance, patient preference for dosage form and acceptability of patient diaries.

**Study design:**

Feasibility double-blind randomised placebo-controlled clinical trial.

**Methods:**

We aimed to recruit 160 patients with cough (≤ 21 days) caused by acute bronchitis from UK general practices. Practices were cluster-randomised to liquid or tablet preparations and patients were individually randomised to Kaloba® or placebo. We followed participants up for 28 days through self-reported patient diaries with telephone support and reviewed medical records at one month. Outcomes included recruitment, withdrawal, safety, reconsultation and symptom diary completion rates. We also assessed treatment adherence, antibiotic prescribing and consumption, mean symptom severity (at days 2–4 after randomisation) and time to symptom resolution. We interviewed 29 patients and 11 health professionals to identify barriers and facilitators to running such a randomised trial.

**Results:**

Of 543 patients screened, 261 were eligible, of whom 134 (51%) were recruited and 103 (77%) returned a completed diary. Overall, 41% (41/100) of patients took antibiotics (Kaloba® liquid group: 48% [15/31]; placebo liquid group: 23% [6/26]; Kaloba® tablet group: 48% [9/21]; placebo tablet group: 50% [11/22]). Most patients adhered to the study medication (median 19 out of 21 doses taken in week 1, IQR 18–21 - all arms combined). There were no serious adverse events relating to treatment. Most patients interviewed found study recruitment to be straightforward, but some found the diary too complex.

**Conclusions:**

It was feasible and acceptable to recruit patients from UK primary care to a double-blind placebo-controlled trial of herbal medicine (Kaloba®) for the treatment of acute bronchitis, with good retention and low data attrition.

**Trial registration:**

HATRIC was registered on the ISRCTN registry (ISRCTN17672884) on 16 August 2018, retrospectively registered. The record can be found at http://www.isrctn.com/ISRCTN17672884.

## Background

Reducing antibiotic resistance is a national and international priority, and the UK aims to reduce antibiotic use in humans in the community by 25% by 2024, from the 2013 baseline [1]. National levels of antibiotic resistance correlate with rates of antibiotic prescriptions [2] and reducing primary care consumption is associated with reduced resistance [3]. At an individual level, risk of antibiotic-resistant infection is over twice as high if an antibiotic has been taken in the past year [4] and this increases the risk of clinical treatment failure [5]. Another important reason to reduce use of antibiotics is the side-effects that they cause; for example nausea, rash and diarrhoea were significantly more common in patients receiving antibiotics for acute bronchitis than in patients receiving placebo [6]. In spite of this, and although they have very little effect on the clinical course of acute bronchitis [6, 7], 82% of patients with acute bronchitis in the UK continue to receive an antibiotic prescription, compared to an “ideal” level of 13% [8].

Several strategies have attempted to reduce inappropriate use of antibiotics. “Delayed” prescriptions (which patients are advised to take only if they are not starting to improve after a certain time) reduce consumption of antibiotics while maintaining patient satisfaction [9], but many patients would still like to take medication to relieve their symptoms because a moderately bad cough is known to persist for around three weeks on average [6, 10]. Commonly recommended treatments like steam and ibuprofen make little or no difference to symptom severity [11]. Other potential symptomatic treatments in adults (the expectorant guaifenesin, mucolytics and antihistamine-decongestant combinations) have not been shown to have consistent benefit in a recently updated Cochrane systematic review [7].

Herbal medicine is another option for relieving symptoms and so reducing inappropriate use of antibiotics [12]. Qualitative research has shown that herbal medicine is widely used and accepted as a viable option for treatment of mild respiratory infections, especially by ethnic minorities, but very few studies have included white Caucasian adults [13–16]. Many patients feel that they need trustworthy advice on whether to use alternative treatments, which to use, and when [17].

Of all the available herbal remedies for respiratory infections, *Pelargonium sidoides* DC (Geraniaceae) is one of the few which already have some evidence of efficacy. It has been used traditionally in South Africa for over a century, both for diarrhoea and for tuberculosis. The first European to “discover” this medicinal plant was Charles Stevens, who was advised by his doctor to go to South Africa after he was diagnosed with tuberculosis. He consulted a traditional healer who treated and cured him with a root decoction [18]. So convinced was he of its effectiveness that he started marketing it in England for treatment of TB in 1904 and it also became widely used in France and Switzerland, where many cases were described of patients with TB who improved after taking this remedy [18, 19]. Stevens kept the identity of the plant secret, and it was not identified until the 1970s [20]. “Umckaloabo” has been marketed in Europe for the treatment of bronchitis since 1991 [20]. It is now widely available over the counter: 990 million defined daily doses of EPs®7630^1^ were placed on the market between 1992 and 2016 (defined daily dose: solution: 4.5 ml, syrup: 11 ml, or 3 tablets, respectively; data from the pharmaceutical manufacturer, 2018).

There are plausible mechanisms to support the potential effect of *Pelargonium sidoides*. Although the root extract EPs® 7630 has only weak antibacterial activity [21] it activates macrophages [22, 23] and reduces binding of group A streptococci to human epithelial cells [24, 25]. It is also active against several respiratory viruses [26, 27] and reduces viral infection of human broncho-epithelial cells by down-regulating cell membrane docking proteins and up-regulating host defence proteins [28]. In mouse and guinea pig models, it has an antitussive effect [29].

Several clinical trials and a Cochrane review have concluded that *Pelargonium sidoides* root extract may be effective at relieving symptoms of acute bronchitis in both adults and children, but the overall quality of the evidence was considered low [30]. Three small randomised controlled trials in acute bronchitis in adults were included and showed inconsistent but overall positive results for resolution of symptoms (all symptoms, cough and sputum production). Three additional studies in acute bronchitis in children were included showing similar inconsistent but positive findings. In adults, symptoms were consistently improved after seven days with an effect observed in individual scale items after 4 days. In a review of the bronchitis severity score (BSS), which included 17 studies of *Pelargonium sidoides* (11 adult and 8 children) a difference in BSS was seen by day 3–5 [31]. The Cochrane review suggested that the liquid formulation may be more effective than the tablets although the number of trials was insufficient to prove this. All the studies included in the review of the BSS showed symptomatic improvement following treatment with *Pelargonium sidoides* regardless of dosage form [31]. *Pelargonium sidoides* tablets are available in the UK and are likely to be more readily available internationally than liquid preparations but it is not clear whether liquid preparations are potentially more efficacious. There is no direct comparative data on tablet and liquid formulations and hence some there is uncertainty over their relative acceptability for consumption.

In the UK, the National Institute for Health and Care Excellence (NICE) suggests that some people may wish to try Pelargonium as a “self-care treatment”, which has “limited evidence of some benefit for the relief of cough symptoms” [32]. There is currently sufficient evidence to recommend the use of *Pelargonium sidoides* root extract to warrant undertaking a high quality independent clinical trial.

The aim of this study was to determine the feasibility of conducting an adequately powered randomised trial of *Pelargonium sidoides* root extract as an alternative to antibiotics for acute bronchitis in UK primary care. The study is called the “HATRIC” trial (Herbal Alternative Treatment for lower Respiratory tract Infections with Cough in adults). A qualitative study, “HATRIC-Q”, was undertaken with a subset of participants and health practitioners involved in the HATRIC trial to explore the feasibility of implementing a full clinical trial and use of herbal medicine to support a delayed antibiotic approach.

## Methods

### HATRIC trial

#### Trial design

We conducted a phase II double-blind feasibility trial with UK GP practices cluster-randomised to give liquid or tablet preparation (for logistical reasons), and within each practice, eligible patients were individually randomised to *Pelargonium sidoides* root extract (EPs®7630 – Kaloba®) or matched placebo. The detailed protocol for this trial has already been published [33]. This trial adheres to CONSORT guidelines.

#### Participants

We recruited patients from 20 GP practices in southern England. We included adults aged 18 years and over, presenting to their GP with an acute cough (≤21 days’ duration) as their main symptom, and with symptoms localising to the lower tract (e.g. sputum, chest pain, dyspnoea, wheeze). This illness definition has been used in other studies in this population [6, 34]. We excluded patients with suspected pneumonia on the basis of focal chest signs (focal crepitations, bronchial breathing) and systemic features (severe breathlessness, high fever, vomiting, severe diarrhoea), patients with serious illness who required hospital admission, exacerbations of COPD, serious comorbidities, and pregnant women (or at risk of pregnancy – defined as any woman of reproductive age not using the combined oral contraceptive pill, a hormonal intrauterine device, a hormonal contraceptive injection, or a subcutaneous hormonal implant).

Eligible patients were invited to participate in the trial by their GP or nurse and were given a patient information sheet during their GP appointment. If they consented, they were then given the study medication and the study diary on the spot and were advised that a trial coordinator would be in touch soon regarding their diary. The randomisation list for sites and patients was generated by Southampton Clinical Trials Unit using the command ralloc in Stata v15 [35]. We used block randomisation (with varying block size) in a 1:1 allocation ratio of placebo to Kaloba® treatment. Treatment packs were sent to sites in sets of four and each patient was allocated the next available sequentially numbered patient pack at their site. Neither the patient nor the doctor or nurse knew to which treatment (Kaloba® or placebo) they had been randomised. Patients were free to withdraw consent from the study at any time without providing a reason.

#### Interventions

Patients on experimental treatment were given a root extract of *Pelargonium sidoides* DC (Geraniaceae). The product used was EPs®7630, manufactured by Dr. Willmar Schwabe GmbH & Co. KG, Germany (named Kaloba®). The extraction solvent used was 11% ethanol (w/w), such that 10 g (= 9.75 mL) of oral solution contains 8.0 g extract from the roots of *Pelargonium sidoides* DC (1: 8–10), and one film-coated tablet contains 20 mg of extract (as dry extract, 1: 8–10). The verum medication provided for the study is taken from production lots sold in the market. All test methods and specifications for the materials, intermediates and the final product are part of the registration dossier and therefore approved by the respective authorities. The liquid also contained glycerol and ethanol (120 mg / 1 ml). The tablets contained the following excipients: Maltodextrin, microcrystalline cellulose, lactose monohydrate, croscarmellose sodium, precipitated silica, magnesium stearate, and the film coating (hypromellose, macrogol, iron oxide yellow E172, iron oxide red E172, titanium dioxide E171, talc, simeticone, methylcellulose and sorbic acid).

The extract was obtained from dried roots of *Pelargonium sidoides* extracted with 11% ethanol w/w, resulting in a drug extract ratio of 1: 8–10 for the liquid extract and the dry extract as well (obtained from the liquid extract by drying). Roots of *Pelargonium sidoides* were collected in South Africa (e.g. Eastern Cape). The dried material was tested in an array of phytochemical and biochemical methods to confirm the quality and identity of the herbal material. Pharmacognosy was done by the quality control department of Dr. Willmar Schwabe GmbH & Co. KG, Germany (Dr. H. Hentrich and lab technicians specifically skilled in pharmacognostic test procedures). A voucher specimen of every lot is deposited in the department of Pharmacognosy to be retained for ten years. In addition, the herbal material is tested with respect to purity (e.g. heavy metals, and microbiological quality).

#### Quality testing

The extract (EPs® 7630) used in the herbal medicinal product (Kaloba® manufactured by Dr. Willmar Schwabe GmbH & Co. KG, Germany) is classified by the European Pharmacopoeia as “other extract” and therefore not adjusted to a particular content of constituents. Independent of this formal classification, the constituents of this herbal active ingredient have been described in detail [36]. Approximately 80% m/m of the extract are assigned to six major groups of constituents, oligomeric prodelphinidines (commonly designated in this context as polyphenols) being the most significant group (approximately 40% of the dried extract).

The liquid herbal medicinal product used in the study (Kaloba® manufactured by Dr. Willmar Schwabe GmbH & Co. KG, Germany) is tested and released compliant with drug GMP and the European Pharmacopoeia according to written, authorized and validated analytical procedures with respect to identity, content (ethanol, glycerin, extract; HPLC) and microbiological quality. The film-coated tablets are tested with the same methods, adapted to the respective dosage form with respect to extract content (HPLC), uniformity, disintegration and microbiological purity. The respective placebo preparation is tested with the same procedures, except for herbal drug content (i.e. absence of active ingredient) and in addition for visual appearance in comparison to the batch of herbal drug product used as medication. The quality testing is performed by the Quality Control laboratory of Dr. Willmar Schwabe GmbH & Co. KG, Germany by a laboratory technician with extensive long-term experience in the testing of herbal medicinal products. Personnel manufacturing or performing quality tests on investigational medicinal products including placebo preparations have been specifically skilled and trained in this field.

The liquid placebo was designed to match the appearance of the liquid herbal medicinal product. This was achieved by using the identical solvent composition (water, glycerol, ethanol) and by replacing the herbal active ingredient with colouring and flavouring. The placebo tablets were also designed to match the appearance of the active tablets, by using the identical film-coating composition and a coloured granulation for manufacturing of the uncoated tablets matching the tablet core of the herbal medicinal product.

The dosage of the liquid (Kaloba® or placebo) was 30 drops (approximately 1.5 ml) three times daily, to be taken 30 min before meals. For tablets (Kaloba® or placebo), the dose was one 20 mg tablet three times daily, to be taken 30 min before meals. Patients were advised to continue this treatment daily until 2–3 days after symptoms had resolved but that treatment duration should not exceed 2 weeks. This is the dosage recommended by the manufacturer and on the traditional herbal registration.

In addition to the trial treatment, GPs were allowed to select the clinically appropriate prescribing strategy as per practice policy, no antibiotics or to prescribe antibiotics, either to be taken immediately, or as a “delayed” prescription.

#### Outcomes

Baseline data were collected at the GP practices where patients were recruited, and medical records were reviewed after one month to extract data on NHS resource usage. Patients were given a diary to complete for 4 weeks, including their symptoms, treatments taken, out-of-pocket expenditure, days off work related to their acute bronchitis and quality of life measurements (EQ5D). All patients were asked to complete the EQ-5D-5L questionnaire at baseline (day 1) and day 7 and approximately half of the patients were also randomly selected to be given the questionnaire at additional time points on days 2 and 4, in order to assess the acceptability of collecting quality of life data more frequently. Patients were telephoned at day 1 or 2 to check for any problems with diary completion, and again at days 14 and 28 days to prompt diary completion and return. If diaries were not returned, or were returned incomplete, a brief telephone interview to collect the key data was undertaken after 35 days.

Feasibility outcomes were: recruitment rate, withdrawal rate from the study, return rate of patient diaries, percentage of completion of patient diaries, compliance with medication according to diary data and returned medication, type of antibiotic prescription given (i.e. immediate/delayed/not given), percentage of patients who took antibiotics, time to antibiotic usage, mean symptom severity (at days 2–4), percentage of patients resolved, time to resolution of symptoms and duration of treatment with herbal medication. The minimum key outcome dataset from the diaries was considered to be [1] antibiotic use in the 28 days post randomisation, [2] no longer experiencing moderate symptoms for two consecutive days and [3] study medication use. A health economic study was a part of the feasibility study which aimed to develop the methods of data collection both for quality of life and for usage of key resources for the design of the future phase III trial.

We explored several possible outcome measures for clinical effectiveness (Table 3). “Duration of symptoms rated as moderately bad or worse” was used in the largest clinical trial of treatment for acute bronchitis [6]. The “last day” definitions allow for the possibility that the illness may fluctuate in severity over the 28-day period and the “first day” definitions do not. Therefore, the last day will tend to be later than or equal to the first day. “Proportion of patients with symptom resolution at day 7” was used in the Cochrane review [30].

#### Statistical methods

The intended sample size was 160 patients overall (40 patients in each of the 4 arms) recruited from 20 GP practices in the UK. No formal sample size calculation was carried out however, ignoring clustering, using a 95% confidence interval approach and an expected proportion of 50% of eligible patients randomised into the trial (to give the worst-case scenario), it can be shown that this sample size would allow us to predict the recruitment rate to within 8% using nQuery Advisor v7.0. Accounting for the clustering based on an intra-cluster correlation (ICC) of 0.05 and an expected proportion of 50% of eligible patients randomised into the trial, this sample size allows us to predict the recruitment rate (number of eligible patients randomised into the trial) to within 13%, given an average cluster size of 8, and 20 recruiting sites.

A detailed statistical analysis plan was developed prior to the analysis. All analyses were conducted using STATA version 15 [35]. We aimed to present descriptive feasibility data rather than to test hypotheses and patients were analysed as randomised.

#### Health economic methods

The economic study was designed from the NHS and a Personal Social Service perspective. In addition, we collected personal costs to test whether a societal perspective should be considered in future trials. Key resources included the costs of the intervention and NHS service use including medication, primary care consultation, outpatient attendance, A&E visits, and hospitalisation. Data on NHS resource usage was extracted through a review of medical records in the 28 days following recruitment. Out-of-pocket spending and days off work related to lower respiratory tract infection were collected through patients’ self-reported diaries. Quality of life data were measured by giving the EQ-5D-5L questionnaire to all patients at baseline (Day 1) and Day 7 and half of the patients were randomly asked to complete the questionnaire at additional time points on Day 2 and Day 4. This aimed to assess the acceptability of more frequently collecting quality of life data in future trials.

### HATRIC-Q nested qualitative study

We conducted a nested qualitative study of patients who met inclusion criteria (including those who did not consent to participate in the clinical trial) and health professionals. Patients were asked for their consent to pass their contact details to the qualitative researcher, who contacted them by telephone to conduct a semi-structured interview, using an interview guide. Health professionals were invited by email. Consent was received verbally by telephone. We aimed for a maximum variation sample, based on pre-specified criteria, such as ethnicity, gender, age, employment status, site, trial drug (liquid drops or tablets), diary status (complete, partly completed, by recall, not returned), and recruitment performance (for the staff interviews). The patients and health professionals who had participated in the trial were asked about trial materials and trial procedures, while those patients who declined to participate were asked for their reasons for non-participation. Interviews were audio-recorded and transcribed verbatim. We also explored the interviewees’ experience of LRTI and its treatment with antibiotics, as well as their attitudes towards delayed prescribing of antibiotics and their views on herbal medicine. Only the results pertinent to trial design are reported here.

Transcripts were analysed using inductive thematic analysis, following the framework approach [37]. The transcripts were compared within and between each other to search for themes, which were then reviewed, defined and named. The analysis followed three key steps:

1. *Familiarization*: repeated readings of transcripts and listening to recordings assisted familiarisation with the data, identification of initial codes and interesting pieces of data
2. *Categorization*: grouping similar responses into meaningful categories
3. *Classification*: assessing relationships between categories and more abstract grouping in order to explain the data

Standard methods were used to safeguard rigor, including multiple coding by DS, RY, MW and GL to check the validity and consistency of coding.

### Ethical and other approvals

HATRIC was registered on the ISRCTN registry (ISRCTN17672884) on 16 August 2018.

## Results

### HATRIC trial – quantitative results

**Fig. 1:**
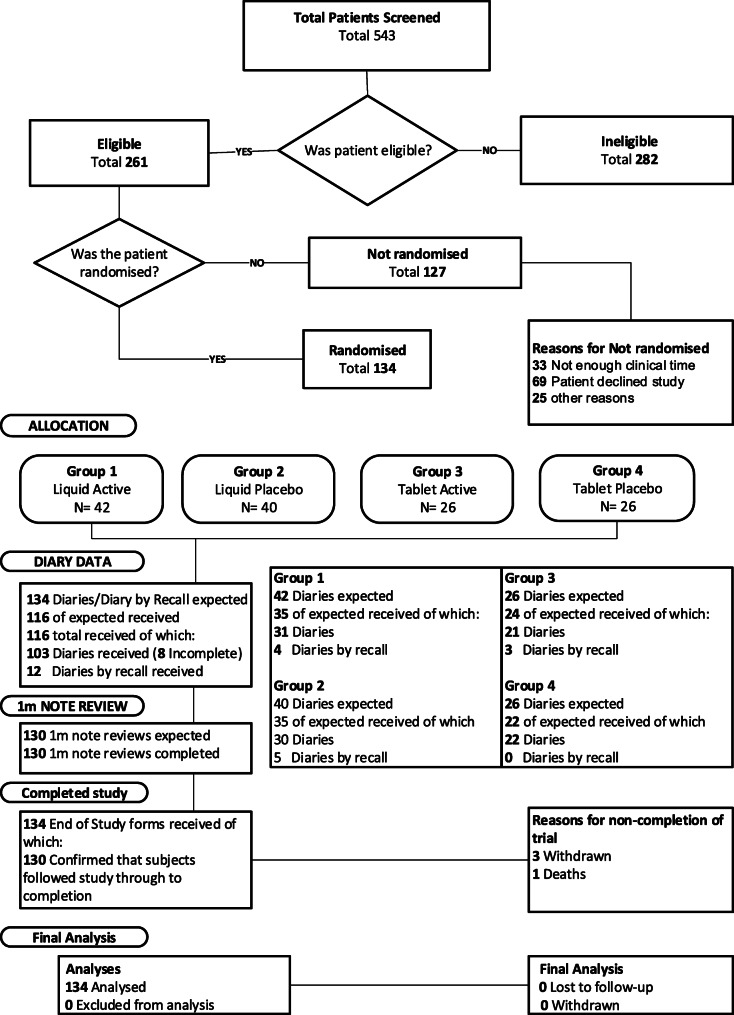
CONSORT diagram

Patients were recruited from March to December 2018, with follow-up ending in January 2019. Five hundred and forty three patients were screened of whom 261 were initially assessed as eligible and 134 (51%) were randomised into the study (Fig. 1). The trial ended after 9 months (rather than the planned 12 months) because it had been 6 months late in starting; over these 9 months it was possible to recruit over 80% of the sample size planned for the 12-month recruitment period.

Of the 127 eligible patients not randomised, in 69 cases this was because the patient declined, and 51 of these gave a reason. Most frequent were concerns about the medication (21/51 patients: 5 didn’t want a placebo; 5 were concerned about potential side-effects; 4 didn’t want to take more medicines; 2 didn’t want a herbal medicine; 2 did not want the liquid preparation; 1 did not want the tablets; 2 wanted antibiotics). Second most frequent (12/51) were patients who were too busy to participate or had personal circumstances which would make it difficult (planned holiday, unwell family member). Nine patients felt either that they were too unwell to take part [6] or that they were improving and didn’t need to take anything [2] or that taking part would make them too anxious [1]. Three patients stated that they just didn’t want to be involved in research. Five patients specifically mentioned that they wouldn’t be good at completing the diary and one would have had to travel to another branch surgery of his GP practice to be recruited.

Of the 134 randomised patients, 82 were allocated to liquid (Kaloba® [*n* = 42] or placebo [*n* = 40]) and 52 to tablets (Kaloba® [*n* = 26] or placebo [n = 26]). During the study period, three patients chose to withdraw – two from the liquid Kaloba® group, one from the liquid placebo group. One patient in the Kaloba® tablet group died due to reasons unrelated to the trial. After completing the trial, four patients were found to have been ineligible because they were women of reproductive age who were using a contraceptive which was not included in the list of the “most effective” contraceptives specified in the protocol. None of these were or subsequently became pregnant during the trial or experienced any adverse events. As the details of their contraception were only confirmed after they had completed the trial, their data is included in the analyses below, as were the data of all the withdrawn patients. The CONSORT diagram is given in Fig. 1.

Recruited patients were primarily white with an average age of 56–59 years (Table 1). Median duration of cough and illness prior to consultation ranged from 6 to 9 days in the different groups. On average, symptom severity at baseline was moderately bad or bad for cough, phlegm and feeling generally unwell. Overall 80/134 (59.7%) were not prescribed an antibiotic, 21/134 (15.7%) were prescribed a delayed antibiotic and 33/134 (24.6%) an immediate antibiotic. The cluster randomisation did not produce a balanced pattern of antibiotic prescription, as practices allocated to the “tablet” groups prescribed more antibiotics (53.8 and 46.2%) than those allocated to the liquid groups, and within the liquid arm, by chance more patients on the Kaloba® liquid received antibiotics (40.4%) than patients on the placebo liquid (27.5%). More of those in the “tablet” groups received delayed prescriptions, whereas in the liquid groups, most received immediate antibiotics. According to their diaries, 41% (40/100) of patients took antibiotics (liquid Kaloba® group: 48% [15/31]; liquid placebo group: 23% [6/26]; tablet Kaloba® group: 48% [9/21]; tablet placebo group: 50% [11/22]).

**Table 1:** Demographic and baseline characteristics of participants

	Liquid Kaloba® (n = 42)	Liquid placebo (n = 40)	Tablet Kaloba® (n = 26)	Tablet placebo (n = 26)	All participants
Female	19/42 (45.2%)	26/40 (65.0%)	15/26 (57.7%)	16/26 (61.5%)	76/134 (56.7%)
Mean age (SD)	56.64 (14.41)	56.65 (17.24)	59.23 (12.31)	57.27 (17.89)	57.27 (15.52)
Ethnicity					
➙White	30/31 (96.8%)	27/27 (100.0%)	20/21 (95.5%)	21/22 (95.5%)	98/101 (97.0%)
➙Mixed	0/31 (0.0%)	0/27 (0.0%)	0/21 (0.0%)	1/22 (4.6%)	1/101 (1.0%)
➙Asian/Asian-British	1/31 (3.2%)	0/27 (0.0%)	0/21 (0.0%)	0/22 (0.0%)	1/101 (1.0%)
➙Prefer not to answer	0/31 (0.0%)	0/27 (0.0%)	1/21 (4.6%)	0/22 (0.0%)	1/101 (1.0%)
Employment					
➙Full/part time	18/31 (58.1%)	13/26 (50.0%)	10/21 (47.6%)	11/22 (50.0%)	52/100 (52.0%)
➙Unable to work	1/31 (3.2%)	1/26 (3.9%)	1/21 (4.8%)	0/22 (0.0%)	3/100 (3.0%)
➙Retired	11/31 (35.5%)	11/26 (42.3%)	10/21 (47.6%)	11/22 (50.0%)	43/100 (43.0%)
➙Full time education	0/31 (0.0%)	1/26 (3.9%)	0/21 (0.0%)	0/22 (0.0%)	1/100 (1.0%)
➙Not working for other reasons	1/31 (3.2%)	0/26 (0.0%)	0/21 (0.0%)	0/22 (0.0%)	1/100 (1.0%)
Smoking habits					
➙Never	15/30 (50.0%)	11/27 (40.7%)	7/21 (33.3%)	10/22 (45.5%)	43/100 (43.0%)
➙Past	14/30 (46.7%)	14/27 (51.9%)	13/21 (61.9%)	8/22 (36.7%)	49/100 (49.0%)
➙Current	1/30 (3.3%)	2/27 (7.4%)	1/21 (4.8%)	4/22 (18.2%)	8/100 (8.0%)
Health conditions					
➙Hypertension	11/42 (26.2%)	9/40 (22.5%)	7/26 (26.9%)	4/26 (15.4%)	31/134 (23.1%)
➙CVD	1/42 (2.4%)	3/40 (7.5%)	0/26 (0.0%)	1/26 (3.9%)	5/134 (3.7%)
➙Lung disease/COPD	0/42 (0%)	1/40 (2.5%)	2/26 (7.7%)	0/26 (0%)	3/134 (2.2%)
➙Diabetes	5/42 (11.9%)	3/40 (7.5%)	3/26 (11.5%)	1/26 (3.9%)	12/134 (9.0%)
➙Asthma	9/42 (21.4%)	8/40 (20.0%)	3/26 (11.5%)	3/26 (11.5%)	23/134 (17.2%)
Antibiotic Prescription					
➙No	25 (59.5%)	29 (72.5%)	12 (46.2%)	14 (53.8%)	80/134 (59.7%)
➙Delayed Use	3 (7.1%)	3 (7.5%)	7 (26.9%)	8 (30.8%)	21/134 (15.7%)
➙Immediate Use	14 (33.3%)	8 (20.0%)	7 (26.9%)	4 (15.4%)	33/134 (24.6%)

1: Symptom severity was scored 0–6 (0 = Normal/not affected; 1 = Very little problem; 2 = Slight problem; 3 = Moderately bad; 4 = Bad; 5 = Very bad; 6 = As bad as it could be)

LQ – Lower quartile; UQ – Upper quartile

Of the 134 randomised patients, 103 returned their diaries (76%) and a further 12 (9%) were completed by recall over the telephone. Initially, 47/103 (46%) of the returned diaries were complete, whereas 56 (54%) had key information missing. These patients were contacted by telephone and key information was completed for an additional 48/54 patients. Therefore, overall we obtained the key outcome dataset for 107/134 patients (80%), while no diary was received for 19 (14%), and incomplete diaries for 8 (6%).

Of 134 participants, 45 (34%) were given EQ-5D-5L at two time points (baseline and 7 days), 89 (66%) were asked to complete it at 4 points (baseline, 2 days, 4 days and 7 days). The completion rates for two points were 69% (31/45) and 67% (30/45) for baseline and 7 days respectively. The completion rates at 4 points in time were 76% (68/89), 76% (68/89), 73% (65/89) and 69% (61/89) at baseline, day 2 and day 4 and day 7, respectively. The completion rates were slightly higher for the group asked to complete EQ-5D-5L at 4 time points. This indicates that more frequent completion of the EQ-5D-5L questionnaire was acceptable in the study population.

Adherence to the study medication proved difficult to measure, because there was not a standard duration of treatment: patients were advised to take the study medication three times daily and to stop 2–3 days after their symptoms resolved (which is subjective). There were no pronounced differences between groups in the proportion who stopped in the first week or two, or who stopped before they had recovered (Table 2). Overall, the median number of doses taken in the first week was 19 (IQR 18–21); ideal would have been 21. This was consistent across the four groups and suggests that treatment adherence was good in all groups.

**Table 2:** Compliance with study medication (total course duration up to 14 days)

	Liquid Kaloba® (***n*** = 27)	Liquid placebo (***n*** = 25)	Tablet Kaloba® (***n*** = 16)	Tablet placebo (***n*** = 20)	All participants
Stopped in week 1	4/27 (14.8%)	5/25 (20.0%)	3/16 (18.8%)	2/20 (10.0%)	14/88 (15.9%)
Stopped in week 2	14/24 (58.3%)	14/20 (70.0%)	9/16 (56.3%)	15/21 (71.4%)	52/81 (64.2%)
Stopped in week 1 but still experienced moderately bad symptoms	2/17 (11.8%)	0/16 (0.0%)	1/9 (11.1%)	1/11 (9.1%)	4/53 (7.5%)
Stopped in week 2 but still experienced moderately bad symptoms	5/9 (55.6%)	6/9 (66.7%)	0/2 (0.0%)	4/7 (57.1%)	15/27 (55.6%)
Stopped in week 1 but had not recovered from all symptoms	2/20 (10.0%)	2/20 (10.0%)	1/14 (7.1%)	2/17 (11.8%)	7/71 (9.9%)
Stopped in week 2 but had not recovered from all symptoms	5/10 (50.0%)	6/10 (60.0%)	1/8 (12.5%)	6/10 (60.0%)	18/38 (47.4%)
Median (IQR) doses taken in week 1	20 (17,21)	18.5 (17,20)	19 (18,21)	19 (18, 21)	19 (18,21)
➙Morning	6 (6,7)	6 (5,6)	6 (6,7)	6 (6,7)	6 (6,7)
➙Afternoon	7 (5,7)	6 (5,7)	7 (6,7)	6.5 (5,7)	7 (5,7)
➙Evening	7 (7,7)	7 (6,7)	7 (6,7)	7 (6,7)	7 (6,7)

This feasibility study was not powered to detect a difference between groups in the time for recovery of symptoms. However, the results appear compatible with the effect size reported in the Cochrane review [30] with those on Kaloba® recovering sooner than those on placebo (Table 3). The last day of all moderately bad symptoms (score 3 or more) was day 7 and 12 in the Kaloba® and placebo liquid groups respectively, and day 9 in both tablet groups. However, these results are based on small numbers and do not control for baseline severity or antibiotic use.

**Table 3:** Symptom severity, duration and medication use from diary

	Liquid Kaloba® (***n*** = 31)	Liquid placebo (n = 26)	Tablet Kaloba® (***n*** = 21)	Tablet placebo (***n*** = 22)	All participants
Last day of any moderately bad symptoms^1^	7 (4,15)	12 (4,17)	9 (5,13)	9 (4,15)	9 (4,14)
First day of no moderately bad symptoms^2^	7 (4,8)	8 (4,13)	6.5 (4,11)	8 (5,14.5)	7 (4,11)
Last day of any symptoms	9 (6,17)	13.5 (7,19)	11 (8,14)	12.5 (8,24)	11 (7,17.5)
First day of no symptoms	10 (6,12)	12 (6,17)	7 (6,12)	11 (8,15)	9 (6,14)
Moderately bad symptoms resolved by day 7?	17/31 (54.8%)	9/26 (34.6%)	9/21 (42.9%)	10/22 (45.5%)	45/97 (46.4%)
Moderately bad symptoms resolved by day 14?	23/31 (74.2%)	17/26 (65.4%)	18/21 (85.7%)	16/22 (72.7%)	74/100 (74.0%)
All symptoms resolved by day 7?	10/31 (32.3%)	7/26 (26.9%)	4/21 (19.1%)	5/22 (22.7%)	26/100 (26.0%)
All symptoms resolved by day 14?	22/31 (71.0%)	15/26 (57.7%)	16/21 (76.2%)	14/22 (63.6%)	67/100 (67.0%)
Antibiotics started?	15/31 (48.4%)	6/26 (23.1%)	9/21 (47.6%)	11/22 (50.0%)	41/100 (41.0%)
Days to first dose of antibiotics in those who reported taking them (median, interquartile range)	1 (1,1)	1 (1,8)	1 (1,9)	1 (1,6)	1 (1,4)

1. The last day of moderately bad symptoms, defined as a score of 3 or more, based on all symptoms recorded in the patient diary

2. The first day without any moderately bad symptoms, a score of 3 or more, based on all symptoms recorded in the patient diary. As severity of symptoms fluctuates, patients may subsequently experience further days with moderately bad symptoms

There were two serious adverse events, neither of which was related to the treatment: a patient in the placebo liquid group suffered a myocardial infarction, and a participant in the Kaloba® tablet group died due to reasons clearly unrelated to the study. Minor adverse events were reported by 7/42 (17%) of patients in the Kaloba® liquid group and in 7/92 (8%) of the other groups (Table 4). The commonest were cough and phlegm which are most likely due to the illness, not to the medication.

**Table 4:** Patient-reported Adverse events

Characteristic	Liquid Kaloba® (n = 42)	Liquid Placebo (n = 40)	Tablet Kaloba® (n = 26)	Tablet Placebo (n = 26)	Total (***n*** = 134)
Number of patients that experienced at least one Side-effect – n (%)^1^	7 (16.7%)	3 (7.5%)	2 (8.0%)	2 (7.7%)	14 (10.5%)
Total number of Side-effects – n^**2**^	28 (68.3%)	8 (19.5%)	3 (7.3%)	2 (4.9%)	41 (100.0%)
Summary of Side-effects – n (%)^2^					
Frequent bouts of Vomiting^3^	1 (3.6%)	0 (0.0%)	0 (0.0%)	0 (0.0%)	1 (2.4%)
Bad taste in mouth	0 (0.0%)	0 (0.0%)	0 (0.0%)	1 (50.0%)	1 (2.4%)
Burping	0 (0.0%)	3 (37.5%)	0 (0.0%)	0 (0.0%)	3 (7.3%)
Cough	4 (14.3%)	0 (0.0%)	0 (0.0%)	0 (0.0%)	4 (9.8%)
Diarrhoea	1 (3.6%)	0 (0.0%)	1 (33.3%)	0 (0.0%)	2 (4.9%)
Disturbed Sleep	1 (3.6%)	0 (0.0%)	0 (0.0%)	0 (0.0%)	1 (2.4%)
Drowsiness	1 (3.6%)	0 (0.0%)	0 (0.0%)	0 (0.0%)	1 (2.4%)
Feeling Bloated	0 (0.0%)	3 (37.5%)	0 (0.0%)	0 (0.0%)	3 (7.3%)
Feeling Unwell	3 (10.7%)	0 (0.0%)	0 (0.0%)	0 (0.0%)	3 (7.3%)
Headaches	3 (10.7%)	1 (12.5%)	0 (0.0%)	0 (0.0%)	4 (9.8%)
Phlegm	5 (17.9%)	0 (0.0%)	0 (0.0%)	0 (0.0%)	5 (12.2%)
Muscle Aches	2 (7.1%)	0 (0.0%)	0 (0.0%)	0 (0.0%)	2 (4.9%)
Nausea^4^	2 (7.1%)	0 (0.0%)	0 (0.0%)	0 (0.0%)	2 (4.9%)
Runny Nose	3 (10.7%)	0 (0.0%)	0 (0.0%)	0 (0.0%)	3 (7.3%)
Stomach Pain	0 (0.0%)	0 (0.0%)	2 (66.6%)	1 (50.0%)	3 (7.3%)
Upset Stomach	0 (0.0%)	1 (12.5%)	0 (0.0%)	0 (0.0%)	1 (2.4%)
Wheeze	2 (7.1%)	0 (0.0%)	0 (0.0%)	0 (0.0%)	2 (4.9%)

^1^ Denominator is the number of patients randomised in the study

^2^ Denominator is number of Side-effects on study

^3^ Patient initially recorded this over all 4 weeks, however related AE only covers period of first week

^4^ One patient recorded nausea in Week 1 diary and Week 2 diary, unsure if this is the same event

All patients had an initial primary care consultation when they were recruited into the study and 22% were reconsulted during the follow-up. A small number (3 in total) used a secondary care service including two outpatient attendances and one hospital admission. 36/134 (27%) of patients reported having used out of pocket purchases and 24/134 (18%) had taken time off work due to their illness. This suggests that a societal perspective should be taken in a future trial.

### Nested qualitative study results

We contacted 43 patients to invite them to be interviewed. Of these, we were able to interview 29 patients, 27 of whom had participated in the HATRIC trial, and two of whom had been invited but declined to participate. We also interviewed 11 health professionals who had been involved in recruiting patients into the HATRIC trial.

### Study recruitment

Barriers and facilitators to study recruitment are summarised in Table 5. Overall the patients reported that the recruitment, information sheet and consent process were straightforward, clear and professional, although a few mentioned that it was too time-consuming. Most participants agreed to take part because they wanted to help the researchers reduce the use of antibiotics, improve their symptoms and trial different options including herbal medicine. However, the interviews revealed that there were a few patients who did not fully understand the purposes and/or some aspects of the trial. One patient would have liked more explanation around the use of antibiotics in relation with the study medication. Another thought that the trial medication ‘*is something that people should be taking sooner rather than later in their illness… in which case I’m not sure whether or not taking it so late … was relevant for your research’*. Some patients explained that recruitment took place when they were feeling poorly, which could have affected their decision. For example, one participant explained that he agreed to take part in the trial to ensure he got antibiotics and could go home. Two patients explained that because their GP would not prescribe antibiotics, they took part because they wanted something for the symptoms. Another assumed the antibiotics were part of the study: ‘*she [GP] just gave them [antibiotics] to me, so I just assumed it’s for the study. That part of it was not particularly well explained’*.

**Table 5:** Facilitators, barriers and suggestions regarding the recruitment process (views of patients in "normal" typeface and views of health professionals in "bold")

Facilitators	Barriers	Suggestions
**(27)** The process was straightforward, clear and professional **(1) well organised packs**	**(4)** Patients did not fully understand the process (outcomes, confused if antibiotics were part of trial) **(3)** Unclear how the results would indicate if Pelargonium is effective when patients are also taking other meds **(7) limited and confusing inclusion criteria (child-bearing age)**	• Better explain the use of antibiotics in relation to the study medication • **have inclusion / exclusion cards**
**(26)** Clinicians’ approach: helpful, informative, reassuring & spent enough time to explain the trial **(2) quick recruitment session** **(1) direct approach in waiting room**	**(4)** Burden: time consuming for GP and patients, paperwork and repetition of consent **(6) time consuming for GPs and nurses** **(2) time consuming for patients** **(3) too much pressure for primary care** **(3) recruitment takes too long**	• consent patients to trial once and remind them that they agreed to be contacted, etc. • warn participants about duration of recruitment appointment • approach patients when feeling better • **reduce paperwork for clinicians** • **research nurses to run the trial and give them protected time** • **condense and simplify process and documents** • **send member of HATRIC team/nurse to discuss with patients before they see the GP** • **run it outside primary care (e.g. A&E, walk-in centres)** • **separate the process (identification –recruitment) and bring back patients** • **remote consent and trial medicine**
**(22)** Clear information on the PIS	**(3)** Fear of side-effects **(6) long diary considered as burden** **(1) severe adverse event**	• recruit patients earlier in their illness • GPs to explain the new drug and if harmless in simple lay terms • **translate PIS in different languages** • **make the font bigger** • **make the diary more user-friendly** • **bound it together better** • **simplify and make it less busy**
**(22)** Patients took part to help with research, reduce Antibiotics use and try alternative ways **(2) patient willing to help with research**	**(3)** Did not understand randomisation **(2) randomisation**	• direct approach by the GP
**(9)** Wish/need to take something when Antibiotics not appropriate **(2)** Prescription of Antibiotics enabled some patients to take part **(3) Offer of (delayed) Antibiotics**	**(2)** If GP had not given them immediate Antibiotics, they would not have considered taking part	• use simple terms and consistently: infection, cough, bronchitis
**(1)** Vouchers were a good incentive **(1) Vouchers are good (but may not work in affluent areas)**	**(5) equipoise (e.g. if too ill, woman of childbearing age)** **(2)** Clinician’s lack of equipoise: (under) report side effects **(1)** GP described the trial as a ‘*secondary thing’*	• use an app for busy people
**(8) patients’ positive views on herbal meds** **(2) staff’s positive views on herbal medicine**	**(4) patients’ negative views on herbal medicine**	
**(5) number of GPs and nurses actively involved in the site**	**(1) not many staff involved**	**train nurses to check eligibility**
**(1) patients’ high demographics**	**(7) seasonal reasons (no eligible patients during summer)** **(4) patients would not bother GP for viral infections** **(1) patients were beyond 3-week period**	**open trial in winter, run full season**
**(2) prompts from the system** **(3) triage system** **(2) introducing trial on the phone** **(2) research-clinical parallel sessions**	**(3) first few recruitments were difficult** **(2) surgery’s system (MAC nurse, multi-site)** **(1) not having** **NHS.net****account to send the forms** **(4) not enough payment for GPs**	• **support the GPs and nurses for recruiting the first 1–2 patients** • **ensure 2nd recruit is soon after the 1st** • **run parallel research – clinical sessions** • **introduce trial when in reception** • **a boost of money to motivate sites monthly competition between sites**

Unfortunately, it proved very difficult to interview patients who did not agree to participate in the trial. The two non-participants explained that they were both initially willing to participate, but one was not eligible and the second declined due to potential side effects (headaches), imminent holiday plans and she was not keen to commit to filling in a daily diary.

From the perspective of the health professionals, the main barriers to recruitment were limiting and confusing inclusion criteria (particularly regarding women of reproductive age – exemplified by the fact that four such women were recruited in error - although they were using a contraceptive, the protocol specified only 4 types of contraception which were admissible); the time taken by the recruitment process; the lengthy patient diary; the lack of patients with relevant symptoms in the summer; and a perception that the payment for GPs to participate in the study was insufficient. The main facilitators to recruitment were patients having a positive attitude towards herbal medicine, and there being several GPs and nurses involved in the recruitment process in the practice. Having a triage system and being able to introduce patients to the idea of the trial before they came for their appointment (over the phone or in the waiting room) also helped, as did the option of offering delayed antibiotics.

### Trial medication

Overall the majority of participants reported that they understood how and when to take the medication and did not encounter any major issues (Table 6). The main difficulties were side-effects and confusion around when to start or stop the study medication. For example, one participant stopped taking the study medication when she started antibiotics because she thought she could not take both together. Another stopped the medication when he felt better and did not realise that he was supposed to carry on for a few more days. Half of the interviewees reported that they did not miss any doses, and most of the others missed just one or two doses (consistent with quantitative results, Table 2). Three patients only took the trial medication for 2–3 days: the first one stopped when he started the antibiotics and the other two when they experienced severe nausea and sickness. Another participant stopped taking the trial medication when he felt better and later started again.

**Table 6:** Facilitators, barriers and suggestions regarding the trial medication (views of patients and health professionals)

Facilitators	Barriers	Suggestions
**(17)** would prefer tablets as are easier to take	**(10)** missed doses	• keep Antibiotics prescription separate from HATRIC folder • enhance the PIS to explain use of Antibiotics and trial meds
**(14)** used strategies to remind them to take the trial medication	**(9)** experienced side effects **(6)** had difficulties with timing (30mins before meals)	• Offer reminding strategies • take medication with or after meal
**(6)** used strategies to dispense the liquid drops	**(6)** had difficulties in dispensing the drops **(3)** had difficulties in measuring the drops	• provide appropriate measure: dripper, small container, teaspoon, top, etc. • better ways of dispensing, explain about shaker bottle • make it more concentrated
**(1)** nurse warned about the taste	**(5)** reported unusual/unpleasant taste	• improve the taste • give only tablets

While most patients taking tablets did not notice a particular taste, half of the participants taking the liquid noticed a particular taste which was described as “*bitter –sour*”, “*unusual fruity*”, “*like it was a root extract… very organic … a nice change”*, “*mildly strange”*, “*something like Bach’s flowers rescue remedy*”, “*evil and gross*”. Patients were asked whether they thought they had taken the real trial medicine or the placebo. About a third of patients thought they might have had the real medicine (because of the unusual taste, because they felt better, or had an adverse effect). About a third of patients thought they might have had the placebo (because they did not feel any improvement in their symptoms). Seven patients had no idea and could not tell either way. Three patients had not understood that they could be given a placebo. “*I am a bit worried about this dummy business. Are you saying you are giving some people pills that you know don’t work*?”. Overall blinding appeared to be successful in the 29 patients included in the qualitative study – only 50% of those on active liquid, and fewer than 50% in the other groups, correctly guessed their allocation, no more than would be expected by chance. Almost all based their guess on whether or not they felt they had improved, rather than on the appearance or taste of the medicine.

Although patients only received one formulation (liquid or tablets) they were asked which they would prefer to receive. Six patients said that they would not mind either. Four patients said that they would prefer the liquid (because there is less pharmaceutical processing involved than in manufacturing tablets, and the liquid felt soothing for the throat). Fifteen said that they would prefer tablets, because they were already taking other tablets, tablets were easy to swallow, they were more convenient, one is less likely to take the wrong dose, there is no problem with spillage, they can be put in a weekly container and they are easier to carry out. The main reason for disliking the liquid was its taste. Several patients reported problems in measuring the liquid dose in drops and thought they may sometimes have taken the wrong dose; they would have preferred to measure the dose using a spoon. Two health professionals expressed similar concerns about dosing the liquid, but one reported that “*I perceived a problem but it wasn’t, it didn’t come to fruition actually… it’s to do with number of drops and things, isn’t it, but it wasn’t a problem*” (HP02, GP).

### Study diaries

The majority of patients said the study diaries were clear, but it was obvious that not all understood or completed them as requested (Table 7). While six health professionals felt the diaries were straightforward, three felt they were too long and confusing, and four told their patients not to take part in the trial unless they could commit to completing the diaries. More than a third of the patients stopped completing their diary prematurely, for example, four when their trial medication finished, and two when they started on antibiotics, because they did not realise that they were expected to continue. Seventeen of the interviewees had needed telephone support to help them understand or complete the diary. Although a majority preferred paper diaries, a small number would have preferred an electronic version. When prompted to discuss several parts of the diaries, some participants mentioned that they found the diary daunting, complex, and somewhat confusing. Some felt confused because of the illness or just felt too sick to complete the diary. The Quality of Life questionnaire (EQ5D) elicited mixed views: almost two thirds of the participants (*n* = 17) reported that it was fine but nine participants found it difficult. Two patients were unsure if it referred to the cough or general health. One said it was “*completely arbitrary*”, another said, “*it’s a very generalised assessment. It all depends whether you’re feeling cheerful on that day for other reasons…”.* Some participants suggested adding more guidance: *“100 means I’m up for running a marathon down to 10 or 20 would be: can’t get out of bed. Something like that, might just be helpful from your point of view to keep things uniform”.*

**Table 7:** Facilitators, barriers and suggestions regarding the trial diaries (views of patients and professionals)

Facilitators	Barriers	Suggestions
[24] Diary was clear and easy [2] diary easy and straightforward [1] pre-paid envelope	[11] found the diary to be daunting, wordy and complex [10] stopped diary prematurely [4] long, repetitive and daunting initially	○ clearer instructions to continue, even when on antibiotics or end of treatment and use big bold font ○ simpler and shorter layout (patients may be unwell) with fewer questions ○ offer yes/no answers ○ do not put tick boxes as people fill them inaccurately ○ change from 1 to 6 to 1–10 as more commonly used ○ could be simpler language ○ make the font bigger ○ make it more user-friendly ○ bind it together better ○ simplify and make it less busy ○ slim down the demographics
[16] EQ5D was easy	[9] found the EQ5D difficult to rate	○ give guidance how to complete EQ5D (e.g. 5 = you don’t have any energy to go out to the shop, 100 = you feel you can walk up a mountain) ○ change EQ5D with a more objective measure, e.g. frequency of coughing, amount & colour of phlegm (easier to remember and report) or ask, ‘*do you feel better today than 2 days ago?’* ○ change EQ5D to 0–10 (too much scope otherwise)
[16] Phone calls from research team were very helpful	[2] missed some sections	○ be clear and remind patients about the trial when the trial team call them ○ more details on early pages on symptoms ○ call /text /email on last day of medicine to remind them to carry on with the diaries
[12] Filling in the diary as you go was helpful		○ follow logical order and split questions to have each day in one page/section (DAY 1 on the top) ○ spread it out more
[8] Diary left in open view as reminder		○ make the days bolder and bigger (not in tiny boxes)
[4] Had a routine		○ offer choice for diaries: paper or electronically

### Overall views on trial procedures

Most interviewed health professionals felt that the trial was well designed and organised (Table 8). A minority felt that payments were insufficient, that the training was too long, and that there were too many forms.

**Table 8:** Barriers and facilitators to trial procedures (views of health professionals)

Facilitators	Barriers	Suggestions
**(8)** Well designed and organised trial **(4)** Well received by patients and clinicians	**(1)** Very difficult trial to run	
**(3)** Storage was fine	**(2)** Moving medicine between sites was difficult **(1)** storing the medicine at the right temperature **(1)** lack of space to store trial paperwork **(1)** handling the medicine when arrived	• provide bigger folders
**(2)** Training was excellent	**(4)** training was too long with too much detail **(1)** training was long time ago	• cascade the training internally • condense the training • iron out the basics online • deliver it nearer the time
**(1**) Electronic database worked well	**(2)** online system difficult to use	• change /review system
**(3)** Very clear paperwork for sites and patients **(1)** coloured folder for the patients	**(3)** too many forms and repetitions	• laminated checklists
**(6)** clear, speedy communication with study team	**(1)** overzealous contacts	
	**(1)** complicated screening **(1)** unsure about screening failures	• only report those who were almost recruited • provide inclusion /exclusion cards
	[4] payment was low **(1)** chasing payments from HATRIC and CRN	
	**(1)** checking returned diaries	• CTU to check diaries
	**(1)** not having a NHS.net email account	• post or fax the paperwork

## Discussion

### Summary of main findings

We were able to recruit 24.7% (134/543) of patients screened for inclusion into this study and 51% (134/261) of those who were eligible. With telephone follow-up it was possible to obtain the key outcome measures for 80%. We recruited 134/160 (84%) of the target sample size however this was over a shorter recruitment period and at a faster rate than predicted (15 patients per month instead of 13). There was a low rate of withdrawal and there were no safety concerns. The cluster randomisation resulted in imbalance between the groups as some GP practices were more likely to prescribe antibiotics. Most patients complied with the medication for at least one week. Most of the patients interviewed were happy with the study procedures and the study diary, although there was some confusion about some sections in the diaries and also whether and when they were expected to take antibiotics. The majority of patients interviewed would have preferred tablets because of the taste and inconvenience of the liquid preparation. Blinding appeared to be successful as only a minority of patients interviewed correctly guessed their allocation to active or placebo. Facilitators to recruitment included patients having a positive attitude towards herbal medicine, a triage system at the GP practice, introduction to the trial before the GP appointment and the option of delayed antibiotics. The main barriers to recruitment were confusion about inclusion criteria, fewer suitable patients during the summer, and perceptions that the recruitment process was time-consuming, that the diary was lengthy, and that the GP payment was insufficient.

### Strengths and limitations

This is the first feasibility trial to be conducted on the use of a herbal medicine for acute bronchitis in UK primary care. A strength of this trial is the use of both quantitative and qualitative methods to explore several feasibility aspects. The trial was not powered to detect effectiveness, so we are unable to comment on the effectiveness of the herbal medicine. However, there was no evidence of any serious side-effects due to the intervention and the patient withdrawal rate was low. Although the study was only open for 9 months as opposed to the 12 months initially planned, we recruited at a faster rate than anticipated.

Limitations included a delay of up to 3 months between patients completing the trial and the qualitative interview, so some interview participants could not recall all details. When asking patients whether they preferred liquid or tablets in the qualitative interviews, each patient had only tried one of these options. They were not aware that the Cochrane review [30] suggests that the liquid may be more effective than tablets; knowing this may have changed their responses.

Although patients suggested changes to the severity of illness rating scale and to the EQ-5D-5L, it will not be possible to make these changes in future trials because these scales are only validated in their current format. Restrictions on which type of contraception were used by women were stringent and were misunderstood by some of the GPs enrolling patients.

### Implications for further research

Our results suggest that progression to a full randomised phase III trial is feasible. The feasibility trial has identified some key modifications which will be needed. It would be easier to choose only one formulation to test (liquid or tablet). Although most patients reported that they would prefer tablets, the Cochrane review [30] suggested that the liquid may be more effective. If the liquid is to be taken into a full trial, a more convenient dosage method may need to be provided as many patients struggled accurately to count 30 drops per dose. If a full study uses both liquid and tablet preparations, we would need to adapt the design to prevent imbalance in the antibiotic use. This could be achieved by individual randomisation (tablet/liquid/active/placebo) or through stratification of practices according to prior antibiotic prescribing history.

As this feasibility study was conducted in GP practices, the future phase III trial should be in the same setting. However, as several patients commented that they would have preferred to start the herbal medicine earlier in their illness there may be potential for a study in pharmacies, to see whether we could also conduct a trial of taking Pelargonium at an earlier stage in the illness, to avoid the need to consult a GP and so to reduce use of antibiotics.

## Conclusions

The HATRIC study showed it is feasible and acceptable to recruit and follow up patients with acute bronchitis from UK primary care into a study of a herbal medicine to facilitate reduction in antibiotic use. Progression to a phase III trial is recommended.

## Data Availability

The datasets used and analysed during the current study are available from the corresponding author on reasonable request.

## Abbreviations

BSS: Bronchitis Severity Score
CTU: Clinical Trials Unit
EQ-5D-5L: EuroQol 5 Dimensions 5 Levels
GP: General Practitioner
HATRIC: **H**erbal **A**lternative **T**reatment for lower **R**espiratory tract **I**nfections with **C**ough in adults
HPLC: High Pressure Liquid Chromatography
MHRA: Medicines and Healthcare products Regulatory Agency
NICE: National Institute for Clinical Excellence
NIHR: National Institute for Health Research
REC: Research Ethics Committee

## Footnotes

1 EPs® 7630 is the active ingredient of the product Kaloba® (ISO Arzneimittel Ettlingen)
